# *Ex Vivo* - Growth Response of Porcine Small Intestinal Bacterial Communities to Pharmacological Doses of Dietary Zinc Oxide

**DOI:** 10.1371/journal.pone.0056405

**Published:** 2013-02-18

**Authors:** Ingo C. Starke, Jürgen Zentek, Wilfried Vahjen

**Affiliations:** Institute of Animal Nutrition, Department of Veterinary Medicine, Freie Universität Berlin, Berlin, Germany; Catalan Institute for Water Research (ICRA), Spain

## Abstract

Piglets were fed diets containing 57 (low) or 2425 (high) mg zinc from analytical grade zinc oxide (ZnO) ·kg^−1^ feed. Digesta samples from the stomach and jejuna of 32, 39, 46 and 53 d old animals (n  = 6 per group) were incubated in media containing 80, 40, 20 and 0 µg·mL^−1^ soluble zinc from ZnO. Turbidity was recorded for 16 h and growth parameters were calculated. Additionally, DNA extracts of selected samples were analyzed via qPCR for different bacterial groups. Samples from animals fed the low dietary zinc concentration always showed highest rate of growth and lowest lag times in media without added zinc. However, media supplemented with zinc displayed highest growth rates and lowest lag time in the high dietary zinc group. Specific growth rates and lag time showed significant differences on day 32 and 39 of age, but rarely on days 46 and 53 of age. Bacterial growth in digesta samples from the high dietary zinc group was less influenced by zinc and recovered growth more rapidly than in the low dietary zinc group. Specific growth rates and bacterial cell numbers from qPCR results showed that lactobacilli were most susceptible to zinc, while bifidobacteria, enterobacteria and enterococci exhibited increased growth rates in samples of animals from the high dietary zinc treatment. No treatment related differences were observed for clostridial cluster IV and the Bacteroides-Prevotella-Porphyromonas cluster. The diversity of enterobacteria after incubation was always higher in the high dietary zinc treatment or in medium supplemented with 80 µg·mL^−1^ soluble ZnO. This study has shown that a pharmacological dosage of ZnO leads to a reduced *ex vivo*- bacterial growth rate of bacteria from the stomach and jejunum of weaned piglets. In view of the rapid bacterial adaptation to dietary zinc, the administration of ZnO in feeds for weaned piglets might only be beneficial in a short period after weaning.

## Introduction

The beneficial effects of pharmacological doses of zinc oxide (ZnO) in animal nutrition, especially piglet nutrition, are well documented [1], [2]. ZnO leads to improved performance and animal health, particularly in the critical period after weaning. The often observed reduction of post-weaning diarrhoea in piglets fed zinc supplemented diets led to the belief that ZnO acts bactericidal. However, relative to the wide-spread use of high dietary zinc levels in animal nutrition, there are surprisingly few *in vivo* studies available on the influence of zinc on intestinal bacteria. In a study by Hojberg et al. [3] especially lactobacilli colony counts were reduced and increased coliform colony counts were observed. Results on bacterial cell numbers from the same animals used in this study also show that Lactobacilli were reduced, but enterobacteria were only reduced in the first week after weaning [4]. Finally, a sequencing study on the effect of high dietary zinc on the microbiota in piglets has shown increased enterobacterial diversity due to high dietary zinc [5].

ZnO has a low water solubility (16 mg L^−1^; solubility product constant: 3.86×10^−10^), but as an amphoteric molecule ZnO displays higher solubility at acidic and alkaline conditions. Due to low stomach pH, ZnO solubility is increased after feed intake and rather high percentages of Zn^2+^ ions can be observed in the stomach of piglets (54% at 164 ppm ZnO/kg diet) [6]. As a consequence, free Zn^2+^ ions may form highly soluble salts with chloride ions and reach the small intestine to act bactericidal.

The stomach and small intestine of piglets harbour a large variety of bacterial species, dominated by lactic acid bacteria, especially lactobacilli [5], [7], [8], [9]. A recent study on the antibacterial activity of ZnO *in vitro* has shown that among the lactic acid bacteria, all tested *Leuconostoc* spp. and *Weissella* spp. were highly resistant, but some *Lactobacillus* spp., *Streptococcus* spp. and *Enterococcus* spp. strains showed lower resistance [10]. Therefore, a shift in the composition of lactic acid bacteria can be expected *in vivo.* In fact, this has been shown for the ileum of 56 d old piglets in a pyrosequencing study [11].

Previous studies on the influence of zinc oxide in weaned piglets focussed on the porcine microbiota at a single time point. No studies are available that tested the sensitivity of bacterial populations against a range of zinc concentrations and their potential to adapt to the presence of dietary zinc at pharmacological dosage. Therefore, this study was conducted to elucidate the effect of zinc oxide on the development of the bacterial growth response and possible adaptation to dietary zinc in the stomach and small intestine of weaned piglets under *ex vivo* conditions.

## Materials and Methods

The study was approved by the local state office of Health and Social Affairs ‘Landesamt für Gesundheit und Soziales, Berlin’ (LaGeSo Reg. Nr. 0347/09).

### Animals and Housing

Purebred landrace piglets were weaned at 26±1 days of age with a mean body weight of 7.2±1.2 kg and randomly allocated into the treatment groups balancing for gender, litter and body weight. Treatment groups were assigned as low dietary zinc (57 mg·kg feed^−1^) and high dietary zinc (2425 mg·kg feed^−1^). Animals were housed in pens (n  = 2 per pen) with straw bedding and *ad libitum* access to feed and water. The room temperature was 26°C at stabling, and was incrementally decreased to 22°C within the first week after weaning according to the standard conditions in the institute. The humidity was kept constant and the light program ensured a 16 hour light and an 8 hour dark phase. No antibiotics were administered before and during the experiment.

### Diets

Piglets received a mash grower diet until 53^th^ d of life (Table S1). The zinc level of the diet was adjusted with ZnO to approximately 50 or 2500 mg zinc per kg feed with analytical grade zinc oxide (Sigma-Aldrich, Taufkirchen, Germany). The dietary zinc levels were confirmed by analysis via atomic absorption spectrometry [12] and yielded 57 and 2425 mg total zinc per kg dry matter.

### Bacteriological Media

A nutrient rich medium (reinforced clostridial medium, LAB022) for cultivation of anaerobic bacteria was used in this study. This medium has been shown to allow growth of a broad range of bacterial strains [10]. Zinc oxide supplemented media were generated by saturating the medium with 10 g analytical grade ZnO (Sigma-Aldrich, Taufkirchen, Germany) per 100 mL medium. After stirring for 30 min at room temperature, the medium was autoclaved (20 min, 121°C, 2 bar) and centrifuged (15 min, 18.500×g, 4°C). Supernatants were collected and total Zn content was determined by atomic absorption spectroscopy [12]. Non supplemented medium was then used to dilute the zinc concentration to 80, 40 and 20 µg·mL^−1^. The zinc supplemented media were prepared fresh one day in advance to each sampling day.

On sampling days, microtiter plate wells (U-form) were filled (200 µl) with the respective freshly prepared media (0, 20, 40, 80 µg·mL^−1^) in a glove box under anaerobic conditions (95% N_2_/5% H_2_).

### Sampling and Incubation

Digesta samples were taken from animals (n  = 6 per group and day) at the age of 32±1, 39±1, 46±1 and 53±1 days of life. After slaughter, individual stomach and mid-jejunum contents were filled into 2 mL plastic tubes and immediately transferred into anaerobic glove boxes. The digesta samples were diluted in zinc free medium (1∶5 vol/vol), mixed gently and left to sediment for 5 min. Supernatants were drawn with cut tips and again diluted in non zinc supplemented medium (1∶10 vol/vol). These sample dilutions were then inoculated in triplicate in prepared microtiter plates, sealed with air tight membranes (Viewseal, Greiner) and transferred into a microtiter plate reader with anaerobic incubation capacity (Tecan Infinite200Pro, Crailsheim, Germany). Non inoculated wells with media served as controls. Plates were incubated for 16 h at 37°C and turbidity was measured at 690 nm every 5 min.

### Calculation of Growth Parameters

Turbidity data and qPCR data were transformed into growth curves and subjected to a nonlinear regression using the logistic sigmoid 3 parameter curve fit equation. Individual growth curves were inspected for goodness of fit and curve fits below r^2^ = 0.98 were rejected from further analysis. Lag times, specific growth rate and maximum growth were calculated for each individual growth curve of the turbidity data. For bacterial cell numbers, some bacterial groups responded in a linear fashion and thus a linear regression was used to determine specific growth rates. To study the growth differences between zinc free and zinc supplemented media of the same digesta sample, the turbidity measurements of incubations in zinc free medium was subtracted from zinc supplemented medium at each time point of the incubation and given as subtractive growth values.

### Determination of Bacterial Cell Numbers from Incubated Samples

#### DNA extraction

Replicate samples of 0 and 80 µg·mL^−1^ zinc media (n = 4 per treatment) were quantitatively removed from microtiter wells after 0, 3, 6, 8 and 16 h incubation and centrifuged (19.000 *g*, 10 min, 37°C). Bacterial cell mass was mixed with 200 µl RNAlater (Qiagen, Hilden, Germany) and frozen at −30°C until further analysis. After thawing, a subsample of 100 µl suspension was used to extract DNA with a commercial DNA extraction kit (Machery-Nagel, Dueren, Germany).

### Realtime PCR - Assays

Primer sequences and annealing temperatures are given in Table S2. All primers were purchased from MWG Biotech (Straubing, Germany). A Stratagene MX3000p (Stratagene, Amsterdam, The Netherlands) was used for PCR amplification and fluorescent data collection. The master mix consisted of 12.5 µL Brilliant II SYBR® Green QPCR Master Mix with Low ROX (Stratagene, Amsterdam, Netherlands), 0.5 µL of each primer (10 µM) and 10.5 µl water. One µl sample was added before PCR amplification. All amplification programs included an initial denaturation step at 95°C for 10 min to activate the polymerase. All PCR programs featured an annealing time of 60 sec and a 60 sec extension at 72°C.

### Quantification of Fluorescent Signals

A detailed description of the quantification procedure is given by Vahjen et al. [13]. In short, overnight cultures from a wide range of bacterial species and known cell numbers (10^9^ cells·mL^−1^) were combined according to their respective phylogenetic groups. After extraction and purification with the same DNA extraction protocol, these extracts were used as PCR calibration samples. Results were expressed as cell number per mL sample.

### Denaturing Gradient Gel Electrophoresis

DNA extracts of the replicates from each sample were pooled and subjected to a qualitative determination of bacterial diversity using the DGGE. PCR was performed with a commercial Multiplex PCR kit (Qiagen, Hilden, Germany). Each PCR mixture contained 0.5 µM of a eubacterial primer pair [14] or a primer pair for enterobacteria developed at our institute, respectively (see Table S2); 100 ng of purified DNA and equal amounts of sterile distilled water and master mix. Amplification of the ribosomal polymerase subunit beta region was carried out in a T1 Thermocycler (Biometra, Göttingen, Germany) with 30 cycles of the following program: initial activation step at 95°C for 10 min followed by a denaturation step at 95°C for 15 sec, an annealing step at 50°C for 1 min, an elongation step at 72°C for 1 min and a final elongation at 72°C for 10 min.

The Ingeny phorU DGGE system (Ingeny, Goes, Netherlands) was used for subsequent nucleotide sequence-specific separation of PCR amplicons using a 30–55% urea gradient in 6% polyacrylamide gels. Electrophoresis was performed at 60°C for 20 h at 100 V. Gels were scanned after silver staining and analyzed by the Phoretix 1D Advanced version V11.2 software package (Nonlinear Dynamics, Newcastle upon Tyne, UK). Richness (number of bands), Shannon index (−∑(P_i_ ln [P_i_]), where P_i_ is the abundance of a species *i* relative to the total number of species) and Evenness (H/ln (S), where H is Shannon index and S is Richness) were calculated from band patterns of the samples. An Unweighted Pair Group Method with Arithmetic Mean (UPGMA) dendrogam was also constructed using the same software.

### Statistical Analysis

Statistical analysis was carried out with SPSS 19.0 (SPSS Inc., Illinois, USA). Arithmetic means and standard deviations were calculated for lag times, specific growth rate, maximum growth as well as specific growth rate and initial as well as final cell numbers for bacterial cell numbers. An unpaired two sample t-test was used to determine statistical significance between zinc concentration regarding lag times, specific growth rate, maximum growth for trial groups, age and intestinal segment as well as for comparison of growth response data after subtraction of turbidity in non zinc supplemented media. Specific growth rates from bacterial cell numbers were analyzed by the non parametric Mann-Whitney-U test. In all cases, a p-value ≤0.05 was considered as significant difference.

## Results

All animals remained clinically healthy during the entire period. Diarrhoea occurred only occasionally with no differences between treatments. The average daily weight gain (ADG) and average daily feed intake (ADFI) were higher (*P*<0.05) in the high dietary zinc group during the first week as compared to the other group, but this effect almost reversed after 3 weeks with higher ADG in the low ZnO group [15].

**Figure 1:**
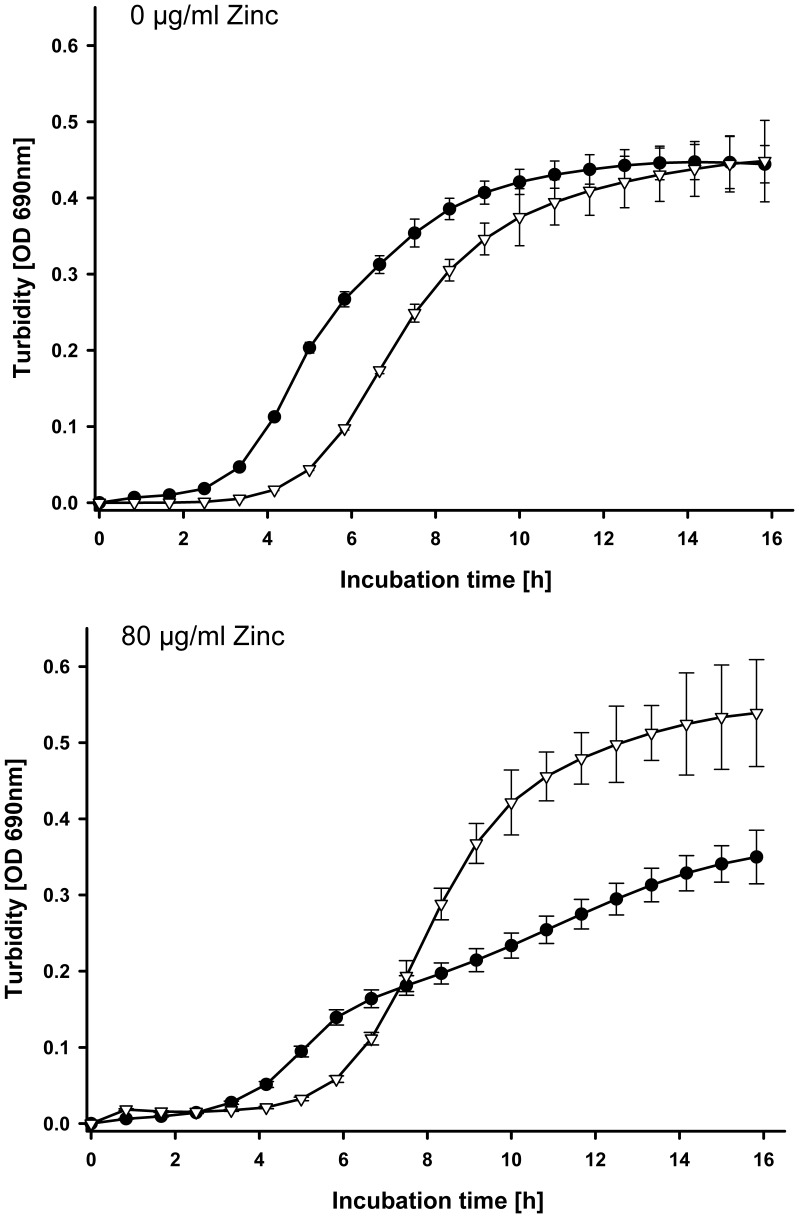
Example for the *ex vivo* growth of bacteria in stomach digesta samples of 32 d old piglets fed low or high dietary zinc in non zinc supplemented medium (•) or medium supplemented with 80 µg zinc·mL^−1^ (Δ).

All incubations in zinc free medium showed typical sigmoid growth curves with varying lag times, exponential growth and stationary phase. Figure 1 displays an example of the *ex vivo* bacterial growth in stomach digesta samples of 32 d old piglets fed low or high dietary zinc when incubated without or with 80 µg total zinc·mL^−1^ medium. In zinc free medium, digesta samples from animals fed the low dietary zinc concentration (57 ppm) showed a shorter lag time, but no higher specific growth rate (ie. slope during exponential growth) than digesta samples from animals fed the high dietary zinc concentration (2425 ppm). Maximum growth after 16 h incubation was identical for both trial groups. At 80 µg·mL^−1^ total zinc in the medium, the lag time of the low dietary zinc group was still shorter, but specific growth rate as well as maximum growth were higher for the high dietary zinc group. This course of growth was also similar in jejunum digesta samples from 32 d old animals, i.e. longer lag times and higher maximum growth as well as higher specific growth rates were observed in the high dietary zinc group. However, significant differences were less frequent than in stomach digesta samples (Table 1). Table 1 also shows that significant differences were more frequent in younger animals, although numerical differences between trial groups still occurred for the 46^th^ and 53^rd^ day of life.

**Table 1:** Influence of dietary zinc on the growth parameters of bacteria in the stomach and jejunum digesta samples of piglets after *ex-vivo* incubation in zinc supplemented media.

			Slope			Max. OD [690 nm]			Lag [h]		
Age	Segment	Zinc [µg/ml]	Low zinc	High zinc	p-value	Low zinc	High zinc	p-value	Low zinc	High zinc	p-value
35	Stomach	0	0.461	0.462	0.963	0.52	0.59	0.109	**3.48^a^**	**4.70^b^**	0.012
		20	**0.387^a^**	**0.491^b^**	0.018	**0.54^a^**	**0.77^b^**	0.026	**3.65^a^**	**5.02^b^**	0.001
		40	**0.355^a^**	**0.548^b^**	0.004	**0.43^a^**	**0.68^b^**	0.000	**4.39^a^**	**5.21^b^**	0.037
		80	**0.310^a^**	**0.652^b^**	0.011	**0.41^a^**	**0.64^b^**	0.007	**5.25^a^**	**5.56^b^**	0.076
	Jejunum	0	0.701	0.746	0.46	0.54	0.61	0.001	4.51	5.17	0.176
		20	**0.608^a^**	**0.769^b^**	0.084	**0.57^a^**	**0.70^b^**	0.004	4.35	4.99	0.414
		40	0.633	0.794	0.351	0.51	0.69	0.129	4.86	5.35	0.552
		80	**0.611^a^**	**0.792^b^**	0.059	**0.46^a^**	**0.67^b^**	0.006	5.17	5.83	0.555
42	Stomach	0	**0.524^a^**	**0.424^b^**	0.018	0.61	0.59	0.008	3.08	4.91	0.006
		20	0.447	0.492	0.156	**0.58^a^**	**0.70^b^**	0.004	**3.07^a^**	**3.75^b^**	0.004
		40	0.413	0.498	0.105	**0.50^a^**	**0.72^b^**	0.005	**3.62^a^**	**4.26^b^**	0.033
		80	**0.383^a^**	**0.494^b^**	0.005	**0.49^a^**	**0.68^b^**	0.023	**4.72^a^**	**5.75^b^**	0.073
	Jejunum	0	0.560	0.616	0.141	0.55	0.47	0.195	**3.23^a^**	**3.77^b^**	0.019
		20	0.515	0.557	0.297	**0.46^a^**	**0.62^b^**	0.081	**3.42^a^**	**4.00^b^**	0.062
		40	0.489	0.562	0.113	0.51	0.66	0.614	3.87	4.24	0.249
		80	0.547	0.649	0.941	0.43	0.61	0.219	4.92	5.21	0.469
49	Stomach	0	0.530	0.480	0.250	0.55	0.52	0.400	4.25	4.94	0.287
		20	0.514	0.482	0.423	0.52	0.58	0.154	4.55	4.29	0.607
		40	0.514	0.500	0.727	0.49	0.54	0.350	4.98	4.53	0.427
		80	0.506	0.556	0.718	0.43	0.48	0.891	5.09	4.94	0.770
	Jejunum	0	0.521	0.508	0.691	0.47	0.45	0.348	4.38	4.40	0.969
		20	0.519	0.482	0.423	0.52	0.54	0.154	4.55	4.29	0.606
		40	0.514	0.500	0.727	0.43	0.48	0.995	4.98	4.53	0.427
		80	0.526	0.536	0.718	0.40	0.52	0.891	5.09	4.94	0.770
56	Stomach	0	0.503	0.453	0.114	0.50	0.58	0.903	**4.79^a^**	**5.09^b^**	0.007
		20	0.487	0.553	0.197	0.52	0.55	0.723	4.68	5.14	0.373
		40	0.456	0.568	0.171	0.50	0.57	0.136	4.83	5.20	0.056
		80	**0.437^a^**	**0.597^b^**	0.031	0.45	0.54	0.909	5.24	5.33	0.851
	Jejunum	0	0.590	0.543	0.140	0.68	0.63	0.196	**4.88^a^**	**5.01^b^**	0.015
		20	0.588	0.643	0.448	0.54	0.57	0.818	4.06	5.07	0.211
		40	0.606	0.663	0.438	0.52	0.58	0.342	**4.47^a^**	**4.21^b^**	0.012
		80	**0.619^a^**	**0.752^b^**	0.001	0.41	0.55	0.64	4.43	4.67	0.124

a, b Means within rows for each parameter with different superscripts differ (*P*<0.05). Significant differences between treatments are highlighted in bold.

In order to evaluate the influence of zinc on the bacterial growth potential in a time dependent manner, turbidity from non zinc supplemented medium was subtracted from respective data of incubations with zinc supplemented media from day 32 to day 53 to yield subtractive growth values. An example for such a growth curve is shown in Figure S1 for samples from the stomach of 32 d old animals incubated medium supplemented with 40 µg·mL^−1^ ZnO. Table 2 shows that bacteria from the high dietary zinc group were less influenced at the highest zinc concentration in the medium and recovered growth more rapidly than bacteria from the low dietary zinc group. For instance, the highest zinc concentration showed significant growth differences on the 32^nd^ day of life from 4 to 10 h incubation in stomach digesta samples and from 6 to 10 h in jejuna digesta samples. Compared to the highest concentration, less influence was visible at 40 µg·mL^−1^ zinc (Table S3). At 20 µg zinc·mL^−1^ medium only a slight numeric growth depression was visible in stomach digesta samples (see Table S3).

**Table 2:** Bacterial growth response to zinc [80 µg·mL^−1^] supplemented medium in stomach and jejunum digesta samples of piglets fed low or high dietary zinc (data after subtraction of turbidity in non zinc supplemented media).

		Stomach			Jejunum			
Day	Time [h]	low zinc	high zinc	p-value	low zinc	high zinc	p-value	
32	2	−0.015	−0.008	0.139	0.001	−0.001	0.756	
	4	−**0.113^a^**	−**0.047^b^**	0.012	−0.051	−0.016	0.084	
	6	−**0.179^a^**	−**0.060^b^**	0.014	−**0.130^a^**	−**0.089^b^**	0.022	
	8	−**0.105^a^**	−**0.011^b^**	0.008	−**0.161^a^**	−**0.038^b^**	0.026	
	10	−**0.013^a^**	**0.009^b^**	0.023	−**0.121^a^**	−**0.057^b^**	0.008	
	12	0.009	0.015	0.264	−0.058	−0.050	0.164	
	14	0.046	0.064	0.235	−0.018	−0.021	0.582	
	16	0.047	0.080	0.101	0.001	−0.004	0.704	
39	2	0.003	−0.002	0.259	0.001	−0.001	0.442	
	4	−**0.078^a^**	−**0.043^b^**	0.041	−0.015	−0.012	0.284	
	6	−**0.164^a^**	−**0.094^b^**	0.005	−**0.113^a^**	−**0.034^b^**	0.044	
	8	−**0.166^a^**	−**0.105^b^**	0.033	−**0.179^a^**	−**0.104^b^**	0.007	
	10	−**0.149^a^**	−**0.095^b^**	0.042	−**0.105^a^**	−**0.043^b^**	0.012	
	12	−**0.118^a^**	−**0.065^b^**	0.009	−0.023	−0.031	0.422	
	14	−0.069	−0.053	0.048	0.033	0.040	0.523	
	16	−0.060	−0.032	0.411	0.064	0.076	0.446	
46	2	0.003	−0.004	0.452	0.007	0.008	0.862	
	4	−0.116	−0.088	0.162	−0.004	−0.001	0.795	
	6	−0.050	−0.035	0.206	−0.060	−0.053	0.387	
	8	−**0.123^a^**	−**0.073^b^**	0.042	−**0.047^a^**	−**0.010^b^**	0.039	
	10	−**0.105^a^**	−**0.062^b^**	0.031	−0.024	−0.009	0.082	
	12	−**0.071^a^**	−**0.030^b^**	0.022	0.039	0.011	0.288	
	14	−0.049	−0.019	0.128	0.050	0.032	0.323	
	16	−0.030	−0.015	0.524	0.064	0.043	0.594	
53	2	0.000	0.007	0.397	0.008	0.005	0.362	
	4	−0.002	−0.004	0.229	−0.007	−0.004	0.114	
	6	−0.033	−0.027	0.172	−0.016	−0.019	0.470	
	8	−0.041	−0.035	0.382	−0.047	−0.024	0.089	
	10	−0.034	−0.025	0.227	−0.024	−0.003	0.084	
	12	−0.018	−0.017	0.693	0.008	0.007	0.824	
	14	−0.015	−0.011	0.552	0.023	0.042	0.611	
	16	−0.020	0.011	0.419	0.045	0.064	0.403	

a, b Means within rows and intestinal segment with different superscripts differ (*P*<0.05, t-test). Significant differences between treatments are highlighted in bold.

Stomach digesta samples from older animals were less influenced by zinc in the high dietary zinc group. Also, growth depression in the low dietary zinc group on the 53^rd^ day of life was less pronounced than on the 46^th^ day of life. A numeric reduction of the growth depression was especially visible for jejuna digesta samples at lower zinc concentrations. However, the low dietary zinc group was also less influenced in older animals.

As the most pronounced differences in bacterial growth behaviour were observed during the first three weeks of the feeding trial, samples from those animals at different time points of the incubation were analyzed for the main bacterial groups present. The specific growth rate of selected bacterial groups as determined from qPCR cell numbers are shown in Table 3. Highest growth rates were generally observed for enterobacteria, followed by bifidobacteria and lactobacilli. Bacteria belonging to the Bacteroides-Prevotella-Porphyromonas cluster did not grow well on all sampling days and intestinal segments, as evidenced by very low to negative growth rates, which followed a linear instead of a logarithmic fashion. Similarly, the clostridial cluster IV showed only linear and low growth rates on 32 and 39 days of life.

**Table 3:** Ex-vivo growth rates of selected bacterial groups in stomach and jejunum samples of piglets fed diets containing 57 ppm (low Zn) or 2425 ppm (high Zn) dietary zinc oxide (n  = 4); significant differences between treatment groups highlighted in bold).

		Stomach				Jejunum			
Age	Bacterial group	0 µg/ml ZnO		80 µg/ml ZnO		0 µg/ml ZnO		80 µg/ml ZnO	
		low Zn	high Zn	low Zn	high Zn	low Zn	high Zn	low Zn	high Zn
32d	Bifidobacteria	**0.148± (0.029)^a^**	**0.203± (0.051)^b^**	**0.145± (0.029)^a^**	**0.195± (0.063)^b^**	0.194± (0.043)	0.219± (0.051)	0.155± (0.047)	0.174± (0.041)
	Bac.-Prevo.-Porphyromonas	−0.088± (0.037)	−0.047± (0.014)	−0.076± (0.039)	−0.028± (0.008)	−0.050± (0.062)	−0.074± (0.021)	−0.037± (0.033)	−0.011± (0.010)
	Clostridium Cluster I	0.132± (0.015)	0.092± (0.080)	**0.155± (0.004)^b^**	**0.088± (0.047)^a^**	0.193± (0.035)	0.172± (0.100)	0.189± (0.015)	0.160± (0.082)
	Clostridium Cluster XIVa	0.080± (0.073)	0.086± (0.007)	**0.071± (0.049)^a^**	**0.139± (0.041)^b^**	0.119± (0.022)	0.104± (0.023)	0.129± (0.003)	0.098± (0.005)
	Clostridium Cluster IV	**0.064± (0.010)^b^**	**0.007± (0.029)^a^**	0.052± (0.004)	0.028± (0.010)	0.032± (0.004)	0.013± (0.037)	0.055± (0.027)	0.021± (0.046)
	Enterobacteria	**0.225± (0.035)^b^**	**0.204± (0.083)^a^**	0.259± (0.011)	0.256± (0.038)	0.247± (0.058)	0.216± (0.051)	0.238± (0.071)	0.251± (0.101)
	Lactobacilli	0.190± (0.005)	0.172± (0.055)	0.148± (0.018)	0.141± (0.063)	**0.248± (0.060)^b^**	**0.174± (0.031)^a^**	**0.218± (0.079)^b^**	**0.155± (0.074)^a^**
	Enterococci	0.105± (0.011)	0.112± (0.068)	0.117± (0.036)	0.185± (0.144)	**0.224± (0.090)^a^**	**0.266± (0.113)^b^**	**0.248± (0.064)^a^**	**0.298± (0.146)^b^**
39d	Bifidobacteria	**0.169± (0.049)^a^**	**0.208± (0.044)^b^**	0.174± (0.054)	0.195± (0.025)	0.226± (0.017)	0.276± (0.006)	**0.218± (0.040)^a^**	**0.279± (0.041)^b^**
	Bac.-Prevo.-Porphyromonas	−0.040± (0.008)	−0.017± (0.027)	−**0.026± (0.004)^a^**	**0.073± (0.026)^b^**	−0.046± (0.025)	0.005± (0.001)	**0.003± (0.019)^a^**	**0.031± (0.026)^b^**
	Clostridium Cluster I	0.105± (0.054)	0.107± (0.020)	**0.127± (0.030)^a^**	**0.148± (0.018)^b^**	**0.063± (0.004)^a^**	**0.108± (0.041)^b^**	**0.104± (0.004)^a^**	**0.156± (0.061)^b^**
	Clostridium Cluster XIVa	**0.005± (0.033)^a^**	**0.076± (0.052)^b^**	**0.006± (0.070)^a^**	**0.106± (0.087)^b^**	0.104± (0.017)	0.086± (0.005)	0.099± (0.002)	0.114± (0.026)
	Clostridium Cluster IV	0.024± (0.090)	−0.018± (0.023)	0.031± (0.042)	−0.028± (0.012)	0.061± (0.029)	−0.047± (0.008)	0.082± (0.019)	0.029± (0.060)
	Enterobacteria	0.176± (0.049)	0.154± (0.004)	**0.189± (0.019)^a^**	**0.235± (0.012)^b^**	**0.188± (0.002)^a^**	**0.231± (0.015)^b^**	0.226± (0.007)	0.240± (0.041)
	Lactobacilli	0.163± (0.015)	0.151± (0.014)	0.129± (0.014)	0.103± (0.024)	**0.225± (0.018)^a^**	**0.153± (0.027)^b^**	**0.171± (0.014)^b^**	**0.129± (0.044)^a^**
	Enterococci	0.083± (0.092)	0.110± (0.079)	**0.107± (0.057)^a^**	**0.134± (0.076)^b^**	**0.141± (0.012)^a^**	**0.204± (0.014)^b^**	**0.225± (0.030)^a^**	**0.259± (0.016)^b^**
46d	Bifidobacteria	**0.168± (0.029)^a^**	**0.217± (0.029)^b^**	0.172± (0.004)	0.198± (0.049)	**0.203± (0.019)^a^**	**0.248± (0.09)^b^**	0.234± (0.085)	0.232± (0.006)
	Bac.-Prevo.-Porphyromonas	−0.016± (0.055)	0.005± (0.001)	0.001± (0.030)	0.058± (0.009)	0.027± (0.025)	0.055± (0.008)	0.037± (0.022)	0.085± (0.025)
	Clostridium Cluster I	0.134± (0.022)	0.153± (0.021)	0.171± (0.018)	0.181± (0.050)	0.120± (0.001)	0.124± (0.008)	0.162± (0.009)	0.178± (0.045)
	Clostridium Cluster XIVa	0.086± (0.021)	0.097± (0.034)	0.115± (0.006)	0.115± (0.018)	0.115± (0.016)	0.117± (0.014)	0.117± (0.044)	0.149± (0.009)
	Clostridium Cluster IV	**0.128± (0.027)^b^**	**0.063± (0.007)^a^**	**0.110± (0.031)^b^**	**0.041± (0.034)^a^**	0.098± (0.057)	0.052± (0.097)	0.06± (0.008)	0.051± (0.089)
	Enterobacteria	0.303± (0.059)	0.304± (0.027)	0.304± (0.020)	0.322± (0.055)	0.303± (0.045)	0.322± (0.077)	0.340± (0.010)	0.352± (0.125)
	Lactobacilli	0.299± (0.038)	0.252± (0.084)	0.271± (0.083)	0.213± (0.103)	0.301± (0.035)	0.256± (0.115)	0.264± (0.056)	0.214± (0.094)
	Enterococci	0.140± (0.037)	0.231± (0.032)	0.165± (0.050)	0.261± (0.048)	0.217± (0.051)	0.266± (0.050)	0.234± (0.069)	0.286± (0.048)

a, b Means within rows for each zinc concentration and intestinal segment with different superscripts differ (*P*<0.05; Mann-Whitney U test).

Bifidobacteria and enterococci showed significantly or numerically increased growth rates in samples from piglets of the high dietary zinc treatment on all sampling days. On the contrary, lactobacilli always showed reduced growth rates in the high dietary zinc treatment. Enterobacteria displayed lower growth rates in samples from the high dietary zinc treatment in non zinc supplemented media, but the same samples exhibited higher growth rates in media supplemented with zinc. The comparison of zinc concentrations in the medium and sample origin also showed that the clostridial cluster I and XIVa showed higher growth rates in media supplemented with zinc on day 39 and day 42 of life, but only on day 32 in stomach samples of the high dietary zinc treatment. Enterococci also seemed to gain a growth advantage, as growth rates at 80 µg·mL^−1^ and in the high dietary zinc treatment were always higher than at 0 µg·mL^−1^ and in the low dietary treatment.

Initial and final cell numbers of selected bacterial groups are shown in Tables S4 and S5. The dominating bacterial groups in non zinc supplemented media after 16 h incubation were lactobacilli, bifidobacteria and enterobacteria. However, lactobacilli already dominated the initial total cell numbers. Compared to non zinc supplemented media decreased lactobacilli counts were observed after 16 h incubation in zinc supplemented media, but bifidobacteria and enterobacteria generally showed a much more pronounced gain in cell number. The increase in cell numbers for other bacterial groups were much less pronounced, especially the Bacteroides-Prevotella-Porphyromonas cluster and the clostridial cluster IV did not increase in cell number in most incubated samples.

**Figure 2:**
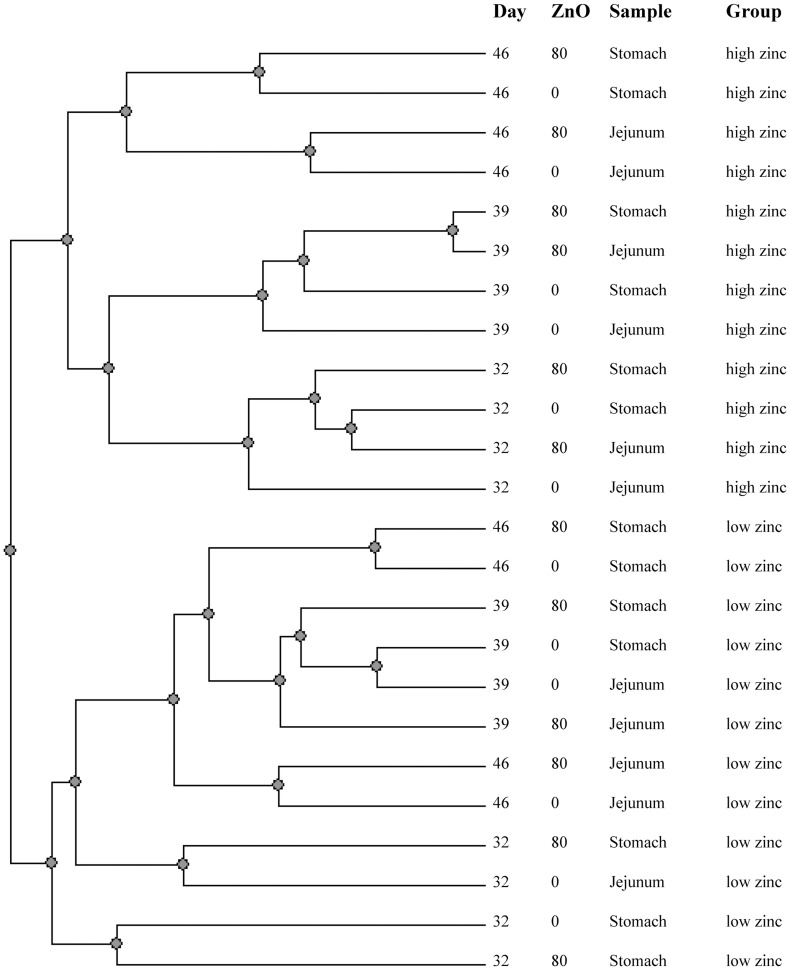
Similarity analysis of DGGE profiles from stomach and jejunum samples of piglets fed low or high dietary zinc after 16 h incubation in control- (0) or zinc supplemented medium (80) (UPGMA method).

The qualitative analysis of the eubacterial composition after 16 h incubation did not show differences between media supplemented with 0 or 80 µg·mL^−1^ zinc (data not shown). However, the similarity of enterobacteria showed a clear clustering for DGGE profiles of sample extracts from the low and high dietary zinc treatment, respectively (Figure 2). Furthermore, all DGGE profiles form the high dietary zinc treatment formed distinct subclusters according to sampling day. This was less evident for profiles from the low dietary zinc treatment. Table 4 shows that the diversity indices for the enterobacterial DGGE profiles were modified due to the presence of zinc in the growth medium. The number of enterobacterial species (ie.richness) was always higher in zinc supplemented medium, regardless of dietary treatment. In turn, the Shannon index showed a higher diversity (except on day 32 in the jejunum of the low dietary zinc treatment).

**Table 4:** Diversity indices for the enterobacterial composition after 16 h incubation of stomach and jejunum samples of piglets fed diets containing 57 ppm (low Zn) or 2425 ppm (high Zn) dietary zinc oxide in media supplemented with 0 or 80 µg·mL^−1^ ZnO.

	Stomach						Jejunum								
	low Zn		high Zn			low Zn				high Zn					
	0 µg Zn	80 µg Zn		0 µg Zn	80 µg Zn			0 µg Zn	80 µg Zn		0 µg Zn	80 µg Zn			
**32 d**															
Richness	4	11		11	13			5	8		13	14			
Shannon	0.86	1.75		1.91	2.02			1.42	1.18		1.99	2.09			
Evenness	0.444	0.665		0.724	0.727			0.682	0.492		0.719	0.737			
**39 d**															
Richness	8	10		5	10			9	14		6	9			
Shannon	1.40	1.66		1.40	1.78			1.39	2.76		1.38	1.72			
Evenness	0.583	0.649		0.675	0.692			0.559	0.937		0.630	0.691			
**46 d**															
Richness	11	13		7	9			7	11		11		14		
Shannon	1.60	1.62		1.48	1.59			0.70	1.65		1.93		2.18		
Evenness	0.605	0.583		0.643	0.641			0.306	0.624		0.695		0.716		

## Discussion

This study used an *ex vivo-*incubation model of gastric and jejuna digesta in complex medium to examine the influence of dietary zinc oxide on the growth potential of porcine stomach and small intestinal bacteria. The use of ZnO supplemented media by centrifugation of saturated media ensured that only soluble ZnO was present during the incubations. Since ZnO solubility increases at acidic pH, the use of ZnO suspensions would have led to increased zinc content at later stages of the incubation due to the formation of volatile fatty acids and lactate. It is however likely that the centrifugation step also removed some proteins as well as phosphates, because it is known that ZnO complexes with peptides or phosphates [16]. Nevertheless, as growth occurred even at the highest zinc concentration, nutrient composition must have been sufficient to support bacterial growth for some bacterial groups. Data from qPCR shows that the Bacteroides-Prevotella-Porphyromonas cluster as well as the Clostridium cluster IV did not grow well under the ex-vivo conditions. This may be due to bacterial competition during the incubation, as enterobacteria also showed low initial cell numbers, but final cell numbers were generally 4 to 5 log units higher.

The lower initial bacterial growth (longer lag time) in digesta from animals fed a high dietary zinc oxide concentration confirms the antibacterial effect of dietary zinc oxide in piglets. This was also visible for initial cell numbers of most bacterial groups, which represents the status quo ante. However, the often observed compensation of growth at the end of the incubation (higher maximum OD) in zinc supplemented media may be due to enhanced growth of a few zinc resistant species. In this experiment, this could be seen for enterobacteria and bifidobacteria, which showed lower initial cell counts than the dominating lactobacilli, but due to a higher growth rate gained relatively more cell mass at the end of the incubation.

The trend for higher maximum OD was only observed in younger animals of the high dietary zinc group and although numerical differences still continued in 46 and 53 day old animals, significant differences were only found for younger animals. Furthermore, specific growth rates (slope of exponential growth) also followed the same trend throughout the feeding trial, as significantly higher specific growth was often observed during the first two weeks of the trial in digesta samples of the high dietary group. Therefore, it is probable that due to reduced competition zinc resistant species rapidly outgrew zinc sensitive species after an initial growth depression. The data from the qPCR analysis suggests that especially bifidobacteria and enterobacteria as well as enterococci were the beneficiary of the reduction of zinc sensitive lactobacilli. Lactobacilli typically dominate the stomach and proximal small intestine of piglets [5], [7], [8], [9] and therefore it is conceivable that a reduction of a dominant group leads to increased growth of other bacteria.

In a recent *in vitro* study, Liedtke and Vahjen [10] tested a broad range of intestinal bacterial strains for their minimal inhibitory concentrations against zinc oxide. That study did not find a clear phylogenetic pattern regarding zinc oxide inhibition, as all bacterial groups showed some members with higher or lower resistance against zinc oxide. The authors concluded that the antibacterial activity of zinc was species specific. Most enterobacteria strains and lactic acid bacteria were not particularly sensitive in that study. Interestingly, *Lactobacillus amylovorus*, one of the dominating *Lactobacillus* spp. in the small intestine of piglets [5], [8] showed the lowest zinc resistance among the tested lactobacilli. This corresponds to results of the *in vivo* study by Hojberg et al. [3] as well as to data for bacterial cell numbers of the same animals [4]. Consequently, it could be speculated for this *ex vivo* study that a reduction of dominant *Lactobacillus* species would leave an increased amount of substrates for zinc resistant lactic acid bacteria or other bacteria, in this case bifidobacteria, enterobacteria and enterococci. This is in agreement with *in vivo* studies that also showed an increased diversity of enterobacteria [5], [17].

The conditions of the *ex vivo* assay led to good growth of enterobacteria and their diversity at the end of the incubation was markedly higher in zinc supplemented media regardless of dietary treatment. Furthermore, DGGE profiles for enterobacteria from the two dietary zinc treatments formed two large distinctly dissimilar clusters. As the enterobacterial composition was different after incubation regardless of zinc content in the medium, the initial distribution of enterobacteria in the samples was probably already modified.

The increased diversity of enterobacteria may be due to reduced bacterial competition during the initial stages, allowing sufficient growth of less prominent enterobacteria. However, another reason behind the increased enterobacterial growth may come from enhanced heavy metal resistance mechanisms, which in general seem to be more efficient in Gram-negative bacteria than in Gram-positive baceria. Gram-negative bacteria rely on specific proton-cation antiporter efflux systems for heavy metals, while Gram-positive bacteria only use P-type efflux ATPases to expel zinc from their cells [18]. Genes for proteins of the Resistance-nodulation-cell-division transporter protein family, specifically for the heavy-metal efflux protein family are much more abundant in gram-negative bacteria than in Gram-positive bacteria [19]. Thus, enterobacteria may possess more efficient systems to expel intracellular zinc.

The enhanced growth of bifidobacteria and enterococci on the other hand may be due to a limited growth capacity of the dominating lactobacilli. As these bacterial groups exhibit a similar physiology, the reduced growth of one group may lead to enhanced growth of the other.

The longer lag phase in incubations of high dietary zinc group samples indicates that either their growth potential or their physiological fitness was reduced in vivo. As this effect diminished in older animals, the degree of resistance to zinc must have increased in bacteria from animals of the high dietary zinc group, ie. an adaptation of those bacterial populations may have occurred during the feeding trial. This was also shown for enterobacteria and *Lactobacillus* spp. in a study, in which the same animals were used to determine bacterial cell numbers [4]. Similar observations have also been reported for microbiota in soil [20]. However, the antibacterial effects of zinc also diminished over time in digesta samples of the low dietary zinc group. Therefore, it can be assumed that due to the development of a more diverse microbiota, an adaptation also occurred in the low dietary zinc group, though at a later time.

### Conclusions

The results of this study have shown that zinc from zinc oxide leads to bacterial growth depression in the stomach and jejunum of weaned piglets in the early phase after weaning. Bacterial adaptation to zinc occurs within 2 to 3 weeks in animals given a diet with a pharmacological zinc oxide dosage. However, bacterial populations in older animals fed a diet with low dietary zinc oxide also seem to adapt to the presence of zinc during *ex vivo* growth. Based on these observations, the administration of ZnO in feeds for weaned piglets seems to be effective only within short periods. Therefore, the use of ZnO could be restricted to the critical time directly after weaning without compromising the beneficial effect on animal health.

## Supporting Information

Figure S1 Detailed display of the bacterial growth response to zinc [80 µg·mL^−1^] supplemented medium in digesta samples of 32 d old piglets fed low or high dietary zinc (data after subtraction of turbidity in non zinc supplemented media).
(TIF)

Table S1 Composition of diets (as-is basis).
(DOC)

Table S2 Primer sequences, product length and annealing temperatures.
(DOC)

Table S3 Bacterial growth response to zinc [40 µg·mL^−1^ and 20 µg·mL^−1^] supplemented medium in stomach and jejunum digesta samples of piglets fed low or high dietary zinc (data after subtraction of turbidity in non zinc supplemented media).
(DOC)

Table S4 Cell numbers of bacterial groups in stomach samples of piglets fed diets containing 57 ppm (low Zn) or 2425 ppm (high Zn) dietary zinc oxide before and after 16 h incubation [log cell number · mL^−1^] (n  = 4).
(DOC)

Table S5 Cell numbers of bacterial groups in jejunum samples of piglets fed diets containing 57 ppm (low Zn) or 2425 ppm (high Zn) dietary zinc oxide before and after 16 h incubation.
(DOC)
