# Supplementary material for: Ex Vivo - Growth Response of Porcine Small Intestinal Bacterial Communities to Pharmacological Doses of Dietary Zinc Oxide
Source: PLoS One. 2013 Feb 18;8(2):e56405. doi: 10.1371/journal.pone.0056405 (PMC3575347; doi:10.1371/journal.pone.0056405)
Supplement: Table S1 — Composition of diets (as-is basis). (DOC) [file pone.0056405.s002.doc]

Supplemental Table S1: Composition of diets (as-is basis)

| Item |  |
| --- | --- |
| Ingredients, g/kg |  |
| Wheat | 380 |
| Barley | 300 |
| Soybean meal | 232 |
| Corn starch/ zinc oxide^1^ | 10 |
| Limestone | 20 |
| Monocalcium phosphate | 20 |
| Mineral & Vitamin Premix^2^ | 15 |
| Soy oil | 17.5 |
| Salt | 2.0 |
| Lysine HCl | 2.5 |
| Methionine | 1.0 |
| Calculated contents |  |
| Dry matter, g/ kg | 879 |
| ME, MJ/kg | 13.0 |
| Crude ash, g/ kg | 81 |
| Crude protein, g/ kg | 194 |
| Crude fiber, g/ kg | 36 |
| Ether extract, g/ kg | 34 |
| Starch, g/ kg | 376 |
| Lysine, g/ kg | 11.7 |
| Methionine, g/ kg | 4.0 |
| Threonine, g/ kg | 7.2 |
| Tryptophane, g/ kg | 2.4 |
| Calcium, g/ kg | 11.0 |
| Phosphorus, g/ kg | 8.0 |
| Sodium, g/ kg | 3.1 |
| Magnesium, g/ kg | 2.2 |
| Zinc, mg/kg^3^ | 34 |
| Iron, mg/kg | 309 |
| Manganese, mg/kg | 81 |
| Copper, mg/kg | 18 |

^1^ Corn starch in the basal diet was partially replaced in the diets containing 50 and 2500 mg/kg zinc with analytical grade zinc oxide (Sigma Aldrich, Taufkirchen, Germany) to adjust for the zinc level.

^2^ Mineral and Vitamin Premix (Spezialfutter Neuruppin Ltd., Neuruppin, Germany), providing per kg feed: 1.95 g Na (as sodium chloride), 0.83 g Mg (as magnesium oxide), 10,500 IU Vitamin A, 1,800 IU Vitamin D3, 120 mg Vitamin E, 4.5 mg Vitamin K3, 3.75 mg Thiamine, 3.75 mg Riboflavine, 6.0 mg Pyridoxine, 30 µg Cobalamine, 37.5 Nicotinic acid, 1.5 mg Folic acid, 375 µg Biotin, 15 mg Pantothenic acid, 1200 mg Choline chloride, 75 mg Fe (as Iron-(II)-carbonate), 15 mg Cu (as Copper-(II)- sulfate), 90 mg Mn (as Manganese-(II)-oxide), 675 µg J (as Calcium-iodate), 525 mg Se (as Sodium-selenite).

^3^ Analyzed concentration of zinc in the basal diet without ZnO supplementation. The other diets contained 57 and 2425 mg/kg, respectively.
