# Supplementary material for: Ex Vivo - Growth Response of Porcine Small Intestinal Bacterial Communities to Pharmacological Doses of Dietary Zinc Oxide
Source: PLoS One. 2013 Feb 18;8(2):e56405. doi: 10.1371/journal.pone.0056405 (PMC3575347; doi:10.1371/journal.pone.0056405)
Supplement: Table S2 — Primer sequences, product length and annealing temperatures. (DOC) [file pone.0056405.s003.doc]

Supplemental Table S2: Primer sequences, product length and annealing temperatures

| **Specificity (target gene)** | **Primer name** | **Sequence** | **Product**  **length[bp]** | **Annealing**  **temperature [°C]** | **Reference** |
| --- | --- | --- | --- | --- | --- |
| *E.coli/ Hafnia/ Shigella* (16S rRNA) | Entero-F | GTTAATACCTTTGCTCATTGA | 340 | 58 | 2 |
|  | Entero-R | ACCAGGGTATCTAATCCTGTT |  |  |  |
| *Enterobacteriaceae* (rpoB) | EntqPCR3417f | GTBTCDCCRCGCAGRC | 435 | 55 | 6 |
|  | EntqPCR3852r | TGCGYCTGGTRATCTA |  |  |  |
| Clostridial-Cluster I (16S rRNA) | CI-F1 | TACCHRAGGAGGAAGCCAC | 231 | 63 | 5 |
|  | CI-R2 | GTTCTTCCTAATCTCTACGCAT |  |  |  |
| Clostridial-Cluster XIVa (16S rRNA) | g-Ccoc-F | AAATGACGGTACCTGACTAA | 440 | 60 | 4 |
|  | g-Ccoc-R | CTTTGAGTTTCATTCTTGCGAA |  |  |  |
| Clostridial-Cluster IV (16S rRNA) | sg-Clept-F | GCACAAGCAGTGGAGT | 239 | 60 | 4 |
|  | sg-Clept-R | CTTCCTCCGTTTTGTCAA |  |  |  |
| *Bacteroides*-*Prevotella* 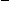 *Porphyromonas* (16S rRNA) | BPP1 | GGTGTCGGCTTAAGTGCCAT | 140 | 55 | 1 |
|  | BPP2 | CGGAYGTAAGGGCCGTGC |  |  |  |
| *Lactobacillus* spp. (16S rRNA) | LAC-1 | AGCAGTAGGGAATCTTCCA | 341 | 58 | 1 |
|  | LAC-2 | CACCGCTACACATGGAG |  |  |  |
| *Enterococcus* spp. (16S rRNA) | Ent1 | CCCTTATTGTTAGTTGCCATCATT | 144 | 61 | 1 |
|  | Ent2 | ACTCGTTGTACTTCCCATTGT |  |  |  |
| *Bifidobacterium* spp. (16S rRNA) | g-BIFID-F | TCGCGTCYGGTGTGAAAG | 243 | 58 | 1 |
|  | g-BIFID-R | CCACATCCAGCRTCCAC |  |  |  |
| DGGE Eubacteria (rpoB) | New rpoB-DGGE (F) | TCA CGG TAA CAA RGG | 431 | 50 | 7 |
|  | New rpoB-DGGE (R) | ^*^AGT GCC CAT ACT TCC AT |  |  |  |
| DGGE Enterobacteria (rpoB) | EntqPCR3417f | GTBTCDCCRCGCAGRC | 435 | 50 | 6 |
|  | EntqPCR3852r | ^*^TGCGYCTGGTRATCTA |  |  |  |

^*^= GC Clamp added: CGCCCGCCGCGCCCCGCGCCCGTCCCGCCGCCCCCGCCCGC

**References**

1 = Rinttilä, T., Kassinen, A., Malinen, E., Krogius, L. & Palva, A. 2004. Development of an extensive set of 16S rDNA-targeted primers for quantification of pathogenic and indigenous bacteria in faecal samples by real-time PCR. Journal of Applied Microbiology  97 (6), 1166-1177

2 = Malinen, E. Kassinen, A. Rinttilä, T. and Palva, A. (2003). Comparison of real-time PCR with SYBR Green I or 5'-nuclease assays and dot-blot hybridization with rDNA-targeted oligonucleotide probes in quantification of selected faecal bacteria Microbiology **149**, 269-277.

3 = Walter J., G. W. Tannock, A. Tilsala-Timisjarvi, S. Rodtong, D. M. Loach, K. Munro and T. Alatossava. 2000. Detection and identification of gastrointestinal Lactobacillus species by using denaturing gradient gel electrophoresis and species-specific PCR primers. Appl. Environ. Microbiol. 66:297-303.

4 = Matsuki T, Watanabe K, Fujimoto J, Takada T, Tanaka R. 2004. Use of 16S rRNA gene-targeted group-specific primers for real-time PCR analysis of predominant bacteria in human feces. Appl Environ Microbiol. 70:7220-7728.

5 = Song Y, Liu C, Finegold SM. 2004. Real-time PCR quantitation of clostridia in feces of autistic children. Appl Environ Microbiol 70:6459-65.

6 = unpublished; developed in the Institute of Animal Nutrition, Free University of Berlin

7 = Perumbakkam S, Craig AM. (2011). Design and in vitro evaluation of new rpoB-DGGE primers for ruminants. FEMS Microbiol Ecol. 76:156-1569
