# Supplementary material for: Ex Vivo - Growth Response of Porcine Small Intestinal Bacterial Communities to Pharmacological Doses of Dietary Zinc Oxide
Source: PLoS One. 2013 Feb 18;8(2):e56405. doi: 10.1371/journal.pone.0056405 (PMC3575347; doi:10.1371/journal.pone.0056405)
Supplement: Table S3 — Bacterial growth response to zinc [40 µg·mL−1 and 20 µg·mL−1] supplemented medium in stomach and jejunum digesta samples of piglets fed low or high dietary zinc (data after subtraction of turbidity in non zinc supplemented media). (DOC) [file pone.0056405.s004.doc]

Supplemental Table S3: Bacterial growth response to zinc [40 µg·mL^-1^and 20 µg·mL^-1^] supplemented medium in stomach and jejunum digesta samples of piglets fed low or high dietary zinc (data after subtraction of turbidity in non zinc supplemented media).

|  |  | 40 µg·mL^-1^ zinc | | | | | | |  | 20 µg·mL^-1^ zinc | | | | | | |
| --- | --- | --- | --- | --- | --- | --- | --- | --- | --- | --- | --- | --- | --- | --- | --- | --- |
|  |  | Stomach | | |  | Jenunum | | |  | Stomach | | |  | Jenunum | | |
| Day | Time [h] | low Zn | high Zn | p-value |  | low Zn | high Zn | p-value |  | low Zn | high Zn | p-value |  | low Zn | high Zn | p-value |
| 32 | 2 | 0.002 | 0.004 | 0.663 |  | 0.001 | 0.002 | 0.516 |  | -0.001 | -0.004 | 0.653 |  | 0.010 | 0.003 | 0. 346 |
|  | 4 | -0.018 | -0.022 | 0.232 |  | -0.005 | -0.002 | 0.843 |  | -0.015 | -0.009 | 0.481 |  | -0.008 | -0.006 | 0.498 |
|  | 6 | -0.077 | -0.042 | 0.072 |  | -0.048 | -0.020 | 0.044 |  | -0.019 | -0.016 | 0.387 |  | -0.006 | -0.011 | 0.178 |
|  | 8 | **-0.098^a^** | **-0.071^b^** | 0.042 |  | **-0.092^a^** | **-0.015^b^** | 0.026 |  | -0.022 | -0.021 | 0.264 |  | -0.019 | -0.021 | 0.348 |
|  | 10 | **-0.131^a^** | **-0.092^b^** | 0.028 |  | -0.094 | -0.064 | 0.079 |  | -0.013 | -0.011 | 0.587 |  | -0.027 | -0.025 | 0.751 |
|  | 12 | -0.110 | -0.086 | 0.322 |  | **-0.087^a^** | **-0.057^b^** | 0.047 |  | -0.005 | -0.009 | 0.115 |  | -0.032 | -0.025 | 0.674 |
|  | 14 | -0.108 | -0.073 | 0.107 |  | **-0.085^a^** | **-0.047^b^** | 0.014 |  | 0.008 | 0.010 | 0.425 |  | -0.031 | -0.022 | 0.379 |
|  | 16 | -0.075 | -0.066 | 0.862 |  | -0.042 | -0.031 | 0.182 |  | 0.008 | 0.012 | 0.611 |  | -0.022 | -0.021 | 0.421 |
| 39 | 2 | -0.005 | -0.003 | 0.473 |  | -0.004 | -0.007 | 0.657 |  | 0.002 | 0.001 | 0.264 |  | 0.005 | 0.006 | 0.822 |
|  | 4 | -0.021 | -0.007 | 0.182 |  | -0.037 | -0.011 | 0.483 |  | -0.03 | -0.007 | 0.814 |  | -0.008 | -0.011 | 0.735 |
|  | 6 | **-0.047** | **-0.010** | 0.047 |  | **-0.122^a^** | **-0.074^b^** | 0.046 |  | -0.058 | -0.051 | 0.185 |  | -0.026 | -0.017 | 0.548 |
|  | 8 | -0.038 | -0.019 | 0.062 |  | **-0.136^a^** | **-0.079^b^** | 0.041 |  | -0.072 | -0.082 | 0.648 |  | -0.047 | -0.037 | 0.199 |
|  | 10 | -0.046 | -0.032 | 0.393 |  | -0.091 | -0.067 | 0.287 |  | -0.068 | -0.071 | 0.228 |  | -0.062 | -0.045 | 0.694 |
|  | 12 | -0.043 | -0.039 | 0.744 |  | -0.082 | -0.045 | 0.154 |  | -0.062 | -0.057 | 0.194 |  | -0.052 | -0.033 | 0.281 |
|  | 14 | -0.038 | -0.034 | 0.502 |  | -0.066 | -0.029 | 0.675 |  | -0.021 | -0.019 | 0.347 |  | -0.041 | -0.023 | 0.106 |
|  | 16 | -0.036 | -0.023 | 0.199 |  | 0.025 | 0.030 | 0.722 |  | -0.014 | -0.008 | 0.365 |  | -0.021 | -0.029 | 0.371 |
| 46 | 2 | 0.008 | 0.007 | 0.283 |  | -0.004 | 0.004 | 0.682 |  | 0.011 | 0.000 | 0.294 |  | -0.003 | -0.002 | 0.455 |
|  | 4 | -0.007 | -0.012 | 0.736 |  | -0.008 | 0.001 | 0.244 |  | -0.001 | -0.004 | 0.844 |  | 0.001 | -0.004 | 0.627 |
|  | 6 | -0.020 | -0.031 | 0.646 |  | -0.049 | -0.018 | 0.673 |  | -0.017 | -0.014 | 0.618 |  | -0.009 | -0.003 | 0.329 |
|  | 8 | -0.059 | -0.070 | 0.209 |  | -0.053 | -0.016 | 0.541 |  | -0.040 | -0.034 | 0.529 |  | -0.014 | -0.013 | 0.405 |
|  | 10 | -0.078 | -0.083 | 0.482 |  | -0.046 | -0.006 | 0.289 |  | -0.029 | -0.031 | 0.374 |  | -0.014 | -0.013 | 0.238 |
|  | 12 | -0.082 | -0.073 | 0.833 |  | -0.033 | -0.006 | 0.415 |  | -0.024 | -0.023 | 0.845 |  | -0.010 | -0.011 | 0.424 |
|  | 14 | -0.071 | -0.057 | 0.252 |  | -0.021 | -0.009 | 0.364 |  | -0.014 | -0.019 | 0.197 |  | -0.010 | -0.012 | 0.642 |
|  | 16 | -0.053 | -0.043 | 0.713 |  | 0.014 | 0.012 | 0.475 |  | -0.008 | -0.011 | 0.649 |  | -0.007 | -0.017 | 0.112 |
| 53 | 2 | 0.002 | 0.002 | 0.773 |  | -0.001 | 0.003 | 0.684 |  | 0.001 | 0.002 | 0.452 |  | 0.003 | 0.002 | 0.681 |
|  | 4 | -0.002 | -0.007 | 0.824 |  | -0.020 | -0.013 | 0.519 |  | -0.002 | -0.009 | 0.501 |  | -0.008 | -0.002 | 0.509 |
|  | 6 | -0.018 | -0.015 | 0.299 |  | -0.048 | -0.035 | 0.643 |  | -0.005 | -0.011 | 0.398 |  | -0.012 | -0.011 | 0.744 |
|  | 8 | -0.020 | -0.005 | 0.658 |  | -0.037 | -0.026 | 0.511 |  | -0.011 | -0.012 | 0.475 |  | -0.015 | -0.016 | 0.298 |
|  | 10 | -0.010 | -0.005 | 0.428 |  | -0.012 | -0.011 | 0.301 |  | -0.020 | -0.022 | 0.388 |  | -0.001 | -0.003 | 0.365 |
|  | 12 | -0.005 | -0.003 | 0.606 |  | 0.007 | 0.015 | 0.284 |  | -0.013 | -0.014 | 0.479 |  | 0.001 | 0.003 | 0.714 |
|  | 14 | -0.001 | 0.001 | 0.241 |  | 0.011 | 0.021 | 0.512 |  | -0.004 | -0.005 | 0.624 |  | 0.005 | 0.006 | 0.542 |
|  | 16 | 0.014 | 0.008 | 0.109 |  | 0.018 | 0.036 | 0.342 |  | 0.007 | 0.012 | 0.571 |  | 0.007 | 0.016 | 0.455 |

^a, b^ Means within a row with different superscripts differ (*P* < 0.05). Significant differences (*P* < 0.05) between treatments are highlighted in bold.
