# Supplementary material for: Ex Vivo - Growth Response of Porcine Small Intestinal Bacterial Communities to Pharmacological Doses of Dietary Zinc Oxide
Source: PLoS One. 2013 Feb 18;8(2):e56405. doi: 10.1371/journal.pone.0056405 (PMC3575347; doi:10.1371/journal.pone.0056405)
Supplement: Table S4 — Cell numbers of bacterial groups in stomach samples of piglets fed diets containing 57 ppm (low Zn) or 2425 ppm (high Zn) dietary zinc oxide before and after 16 h incubation [log cell number · mL−1] (n = 4). (DOC) [file pone.0056405.s005.doc]

Supplemental Table S4: Cell numbers of bacterial groups in stomach samples of piglets fed diets containing 57 ppm (low Zn) or 2425 ppm (high Zn) dietary zinc oxide before and after 16h incubation [log cell number · mL^-1^] (n = 4)

|  | Incubation time | 0h | | 16h | | | |
| --- | --- | --- | --- | --- | --- | --- | --- |
|  | Zinc in medium | - | | 0 µg/ml ZnO | | 80 µg/ml ZnO | |
|  | Treatment group | low Zn | high Zn | low Zn | high Zn | low Zn | high Zn |
| 32d | Bifidobacteria | 6.7 ± (0.4) | 5.9 ± (1.2) | 9.2 ± (1.0) | 9.2 ± (0.5) | 9.1 ± (0.9) | 9.1 ± (0.3) |
|  | Bac.-Prevo.-Porphyromonas | 6.4 ± (0.2) | 6.1 ± (0.6) | 4.9 ± (0.4) | 5.2 ± (0.4) | 4.9 ± (0.5) | 5.4 ± (0.7) |
|  | Clostridium Cluster I | 4.9 ± (0.2) | 5.8 ± (0.3) | 6.8 ± (0.3) | 7.2 ± (1.8) | 7.2 ± (0.2) | 7.1 ± (1.3) |
|  | Clostridium Cluster XIVa | 6.2 ± (1.1) | 5.8 ± (0.1) | 7.4 ± (0.2) | 7.0 ± (0.2) | 7.4 ± (0.3) | 7.8 ± (0.3) |
|  | Clostridium Cluster IV | 4.1 ± (0.2) | 4.6 ± (0.2) | 4.8 ± (0.2) | 4.7 ± (0.7) | 4.9 ± (0.2) | 5.0 ± (0.3) |
|  | Enterobacteria | 4.6 ± (0.5) | 5.3 ± (0.1) | 8.5 ± (0.1) | 8.9 ± (1.1) | 9.1 ± (0.4) | 9.8 ± (0.6) |
|  | Lactobacilli | 7.0 ± (0.2) | 7.4 ± (0.8) | 10.3 ± (0.2) | 10.4 ± (0.1) | 9.7 ± (0.2) | 9.7 ± (0.2) |
|  | Enterococci | 4.4 ± (0.1) | 4.6 ± (0.2) | 6.0 ± (0.4) | 6.5 ± (1.5) | 6.3 ± (0.5) | 7.6 ± (2.7) |
|  |  |  |  |  |  |  |  |
| 39d | Bifidobacteria | 7.0 ± (0.4) | 6.2 ± (1.1) | 9.8 ± (0.3) | 9.4 ± (0.6) | 9.9 ± (0.4) | 9.1 ± (0.8) |
|  | Bac.-Prevo.-Porphyromonas | 6.5 ± (0.1) | 5.9 ± (0.9) | 5.6 ± (0.1) | 5.5 ± (0.5) | 5.9 ± (0.1) | 6.9 ± (0.5) |
|  | Clostridium Cluster I | 6.0 ± (0.4) | 5.9 ± (0.5) | 7.4 ± (0.1) | 7.5 ± (0.2) | 7.7 ± (0.2) | 8.1 ± (0.1) |
|  | Clostridium Cluster XIVa | 7.7 ± (0.6) | 6.1 ± (1.3) | 7.3 ± (0.2) | 7.2 ± (0.6) | 7.5 ± (0.4) | 7.7 ± (0.1) |
|  | Clostridium Cluster IV | 5.1 ± (0.1) | 4.5 ± (0.5) | 4.7 ± (0.6) | 4.5 ± (0.7) | 4.6 ± (0.5) | 4.9 ± (0.2) |
|  | Enterobacteria | 5.6 ± (0.1) | 5.6 ± (0.6) | 8.6 ± (0.2) | 9.1 ± (1.2) | 9.1 ± (0.1) | 9.8 ± (0.4) |
|  | Lactobacilli | 8.0 ± (0.2) | 7.6 ± (0.2) | 10.6 ± (0.1) | 10.5 ± (0.1) | 9.7 ± (0.1) | 9.7 ± (0.3) |
|  | Enterococci | 5.1 ± (0.2) | 5.1 ± (0.3) | 6.0 ± (1.4) | 6.5 ± (0.9) | 7.0 ± (1.5) | 6.7 ± (0.7) |
|  |  |  |  |  |  |  |  |
| 46d | Bifidobacteria | 4.9 ± (1.3) | 5.3 ± (0.3) | 8.4 ± (0.9) | 8.0 ± (0.1) | 8.1 ± (0.5) | 8.2 ± (0.1) |
|  | Bac.-Prevo.-Porphyromonas | 5.6 ± (0.9) | 6.1 ± (0.1) | 5.1 ± (0.1) | 5.8 ± (0.2) | 5.3 ± (0.6) | 6.7 ± (0.1) |
|  | Clostridium Cluster I | 4.0 ± (0.2) | 4.7 ± (0.4) | 6.1 ± (0.3) | 7.1 ± (0.2) | 6.7 ± (0.2) | 7.6 ± (0.4) |
|  | Clostridium Cluster XIVa | 5.3 ± (0.4) | 5.7 ± (0.3) | 6.7 ± (0.2) | 7.2 ± (0.3) | 7.2 ± (0.6) | 7.7 ± (0.1) |
|  | Clostridium Cluster IV | 3.9 ± (0.7) | 4.5 ± (0.4) | 6.0 ± (0.2) | 5.3 ± (0.2) | 5.8 ± (0.1) | 5.2 ± (0.2) |
|  | Enterobacteria | 3.9 ± (0.1) | 4.4 ± (0.1) | 9.1 ± (1.0) | 9.2 ± (0.5) | 9.2 ± (0.7) | 9.4 ± (0.3) |
|  | Lactobacilli | 5.7 ± (1.5) | 5.1 ± (0.5) | 10.1 ± (0.1) | 10.3 ± (0) | 9.1 ± (0.1) | 9.6 ± (0.5) |
|  | Enterococci | 4.5 ± (0.1) | 5.2 ± (0.6) | 8.2 ± (0.7) | 7.4 ± (1.4) | 8.6 ± (0.8) | 7.9 ± (1.4) |
